# Care challenges and silver linings in HIV and behavioral health service delivery for individuals living with HIV and severe mental illness during the COVID-19 pandemic: a qualitative study

**DOI:** 10.1186/s12913-024-11146-1

**Published:** 2024-05-31

**Authors:** Priya Dahiya, Nicholas S. Riano, James W. Dilley, Mark Olfson, Francine Cournos, Christina Mangurian, Emily A. Arnold

**Affiliations:** 1 Department of Psychiatry & Behavioral Sciences, Weill Institute for Neurosciences, University of California San Francisco, School of Medicine, 675 18th St, San Francisco, CA 94143 USA; 2 Department of Psychological Science, School of Social Ecology, University of California Irvine. 4220 Social and Behavioral Sciences Gateway, Irvine, CA 92697 USA; 3 Department of Epidemiology & Biostatistics, University of California San Francisco, School of Medicine, 550 16th Street, Second Floor, San Francisco, CA 94158 USA; 4 Center for Vulnerable Populations, Zuckerberg San Francisco General Hospital, 2789 25th Street, Suite 350, San Francisco, CA 94110 USA; 5 New York State Psychiatric Institute, 1051 Riverside Drive, New York, NY 10032 USA; 6 Department of Psychiatry, Columbia University Vagelos College of Physicians and Surgeons, 630 West 168th St, New York, NY 10032 USA; 7 Center for AIDS Prevention Studies, University of California San Francisco, 550 16th Street, 3rd Floor, Box 0886, San Francisco, CA 94143 USA

**Keywords:** COVID-19, HIV, Behavioral health, Healthcare delivery, Severe mental illness, Telehealth, Workforce burnout

## Abstract

**Background:**

There has been a longstanding effort to integrate behavioral health and HIV care for people with comorbid HIV and behavioral health needs, including those with severe mental illness (SMI). As this population frequents both behavioral health and HIV care settings, they were likely to experience new obstacles to the quality and availability of care during the COVID-19 pandemic. This study aims to describe how clinics for HIV services or behavioral healthcare—as well as co-located sites providing both—sought to rapidly shift protocols to maintain a standard of patient care for people with comorbid HIV and SMI while adapting to the unprecedented circumstances of the pandemic.

**Methods:**

We interviewed HIV and behavioral healthcare providers, clinic leaders, and support service agencies that served clients impacted by both HIV and SMI. Seventeen key informants across three settings (HIV care settings, behavioral health care settings, and integrated or co-located care settings) were interviewed in 2022. Interviews focused on changes in clinical services, protocols, and care provision strategies during and at the onset of the COVID-19 pandemic. Interviews were transcribed and coded using thematic analysis.

**Results:**

Commonly endorsed themes included both positive and negative changes in care and care provision during the pandemic. Negative impacts of the pandemic included the loss of physical space, exacerbated mental health needs and disengagement in HIV care, patient barriers to telehealth and the digital divide, and increased healthcare workforce burnout. Positive changes included improved healthcare delivery and care engagement through telehealth, new opportunities to provide a wide range of social services, paradoxical increases in engagement in HIV care for certain patients, and broad institution of workforce wellness practices.

**Conclusions:**

Though COVID-19 presented several complex barriers to care for providers serving patients with comorbid HIV and SMI, the increased flexibility afforded by telehealth and a greater focus on collaborative approaches to patient care may benefit this patient population in the future. Additionally, the focus on workforce wellness may serve to increase retention and avoid burnout among providers. The strategies and lessons learned through adapting to COVID-19 may be invaluable moving forward as healthcare systems respond to future pandemics.

**Supplementary Information:**

The online version contains supplementary material available at 10.1186/s12913-024-11146-1.

## Background

COVID-19 restrictions have significantly affected health care delivery across the United States, exacerbating existing issues and reducing the accessibility of many health and social services [1]. The systemic changes made to behavioral health services and HIV care at the onset of the pandemic have created new obstacles to care that disproportionately affect vulnerable populations. One such population is people living with HIV (PLWH) and comorbid severe mental illness (SMI, e.g., schizophrenia, schizoaffective disorder, bipolar disorder, chronic major depressive disorder), who are the main population served in public behavioral health care systems [2–4].

Across the HIV treatment cascade, mental illness has been associated with poorer outcomes overall, but individuals with SMI in particular have low rates of HIV testing [5], antiretroviral therapy prescriptions [6, 7], and viral suppression when compared to the general HIV population [8, 9]. Moreover, prior improvements in care across the HIV care continuum (e.g., improvements in viral suppression) have slowed due to the COVID-19 pandemic [10], making it possible that vulnerable sub-populations of PLWH have experienced even further setbacks. Despite these disparities, few studies have explored the role of COVID on accessing both HIV and behavioral health care for PLWH and comorbid behavioral health issues, particularly SMI.

In terms of the impact of COVID-19 on HIV care settings, COVID-19 prevention and mitigation efforts such as statewide shelter-in-place (SIP) mandates [11], social distancing [12], and other location-specific restrictions, in addition to the closure of HIV programs or decreased service offerings [13], all presented challenges to the HIV care continuum, complicating access to HIV testing, prevention services, and treatment [14]. While new HIV diagnoses in the United States decreased by 17% between 2019 and 2020, the Centers for Disease Control and Prevention (CDC) noted that this decrease was likely spurious, driven by disruptions in HIV services and testing related to the pandemic [15]. For example, following its SIP mandate, San Francisco reported a 40% decline in health facility HIV testing, in addition to a decrease in viral load monitoring and in frequency of pre-exposure prophylaxis (PrEP) visits [16]. These declines continued beyond 2020, as HIV testing rates have yet to return to pre-pandemic levels [17–19]. 

One study exploring the impact of COVID-19 on HIV service delivery in California found that while clinics with flexible funding streams were able to adapt to COVID-19 mandates, some struggled to maintain pre-COVID service levels [20]. Additionally, many HIV serving clinics faced new challenges in identifying, engaging, and retaining PLWH without in-person services [20]. For example, some struggled to obtain the necessary technology for telehealth for all patients [21], or to procure timely refills [22], ultimately jeopardizing HIV treatment adherence and widening care disparities [23]. Relatedly, clinics within the San Francisco Department of Public Health (SFDPH) reported that 53% of people on PrEP described challenges with medication adherence following COVID-19 restrictions due to healthcare access problems, including the inability to access laboratories, receive PrEP refills, communicate with providers, or make clinic appointments [24]. This study also reported that over half of these individuals reported worsened quality of life and mental health following the SIP mandate [24]. Still another study reported poorer overall HIV self-management [25].

COVID-19 also posed additional challenges to SMI patients within the behavioral health care system. Already known to face significant challenges in engaging and maintaining these patients in care, SMI patients were especially challenged by the shift to telehealth due to technological difficulties, patient disinterest and challenges with managing remote access via platforms such as Zoom, and attrition [26]. Additionally, conducting comprehensive mental health assessments and facilitating patient connection to care became more difficult, as providers found holistic interpretation of patient symptoms more challenging when screenings were held virtually. Furthermore, for HIV clinics wanting to refer such patients to mental health clinics, referrals to these outside services were more difficult due to overall increased demand [27]. Finally, the need for behavioral healthcare expanded rapidly during the pandemic; many individuals sought services for the first time due to downstream consequences of shelter-in-place and social distancing [28]. This served to increase the behavioral health patient population during a decrease of service availability, taxing the behavioral health care workforce, and the larger healthcare system overall [29]. 

For people with HIV and mental health needs, reports have focused on an increased demand for mental health services during the COVID-19 pandemic. For example, a study of PLWH receiving care at the Vanderbilt Comprehensive Care Clinic reported an increase in mental health encounters by 14%, an increase in mental health electronic communications by 60%, and an increase in mental-health-provider-initiated medication refills by 20% [30]. University of Chicago Medicine also reported increased engagement in behavioral health services among PLWH without prior history of receiving these services [31]. This increased need for mental health support also extended to the general healthcare workforce, who experienced higher rates of burnout and poor mental health [29, 32, 33]. 

People living with HIV and SMI have been particularly affected by the systemic changes to healthcare delivery during the COVID-19 pandemic given that this population often accesses a combination of both HIV and behavioral health care delivery systems, yet there is a dearth of existing literature that documents these challenges. As such, this study aims to understand how the HIV and behavioral health care systems (as well as co-located care sites providing both types of services) adapted to COVID-19 policies and unprecedented circumstances to maintain access to care and meet patient needs during the first two years of the pandemic. By exploring multiple adjustments to daily clinical protocols and innovative care engagement efforts, this study reflects on both challenges and barriers to care, as well as opportunities or silver linings that ultimately enhanced access to care for this population.

## Methods

This qualitative, exploratory study was conducted with health care providers, clinic leaders and representatives from support service agencies serving clients impacted by HIV and SMI. We conducted interviews intentionally sampling to gain deeper understandings of the settings where this population typically received care [34, 35], aiming to include perspectives from both HIV as well as public behavioral health care organizations and clinics in California, including those who worked in settings where HIV and behavioral health care services were co-located and integrated. California is a state with a high prevalence of HIV. California’s public behavioral health care system primarily serves people with SMI and is decentralized—with each county designing their own system of care. Thus, we directly contacted representatives from organizations using networks preestablished through other policy-related research [20, 36, 37], as well as through clinic/agency websites in order to represent different types of agencies and areas of the state, including clinics from Northern California, Southern California, and the Central Valley. A total of 27 agencies were approached and 17 agreed to participate (7 were HIV and behavioral health focused, 8 were HIV-focused, 2 were solely behavioral health focused). Data collection continued until our understandings of the challenges and opportunities that COVID presented to HIV and behavioral health care providers were rich, meaningful, and nuanced. Our team reviewed transcripts and interview summaries and noted areas for further refinement while we were actively collecting data, allowing us to intentionally sample for additional perspectives where thematic areas were under described [38]. 

From February through September 2022, a total of 17 key informants were interviewed (see Table 1). Interview topics included clinic services, changes to protocols and services following onset of COVID in March 2020, efforts to maintain HIV testing and care engagement and/or maintain access to mental health services, the role of telehealth and remote work, and the role of electronic medical data systems and integrated care. Interviews were conducted by trained, seasoned qualitative interviewers (EA and JWD), and lasted between 45 and 75 min. Please see Appendix A for a copy of the interview guide, which was developed specifically for this study. Interviews took place on Zoom, were audio recorded, and professionally transcribed. Participants received the study information sheet by email in advance of the interview and provided verbal informed consent at the beginning of the study activity. All participants were interviewed individually and were offered a $100.00 honorarium in the form of a gift card or a check at the conclusion of the interview. The Institutional Review Board at the University of California San Francisco reviewed and approved all study procedures (UCSF IRB Study # 21-35073).

**Table 1:** Key Informant Sites and Titles

Key informant	Organizational/clinical focus	Role
KI01	Integrated HIV and Behavioral Health	Clinic Administrator and Behavioral Health Provider
KI02	Integrated HIV and Behavioral Health	Clinic Administrator and Behavioral Health Provider
KI03	Integrated HIV and Behavioral Health	Social Worker/Program Manager
KI04	HIV	HIV Provider
KI05	HIV	Behavioral Health Provider
KI06	HIV	Provider and Social Worker/Program Manager
KI07	HIV	Clinic Administrator
KI08	Behavioral Health	Clinic Administrator and Behavioral Health Provider
KI09	HIV	Clinic Administrator and Provider
KI10	Integrated HIV and Behavioral Health	Social Worker/Program Manager
KI11	Behavioral Health	Clinic Administrator
KI12	HIV	Clinic Administrator and Provider
KI13	Integrated HIV and Behavioral Health	Clinic Administrator
KI14	HIV	HIV Provider
KI15	HIV	Clinic Administrator
KI16	Integrated HIV and Behavioral Health	Clinic Administrator
KI17	Integrated HIV and Behavioral Health	Social Worker/Program Manager

Following the tenets of thematic analysis [34], the study team developed a coding scheme based on deductive themes within the interview guide as well as unanticipated, inductive themes. The coding scheme was tested and refined, where all members of the analytic team applied the codes to a subset of 5 interviews to ensure narratives were correctly captured. Then, the interview transcripts and coding scheme were entered into Dedoose, a qualitative analytic program, and all 17 transcripts were coded by primary and secondary analysts. Because the team was interested in documenting the unique barriers to HIV prevention and care that people with SMI experienced during COVID, and the various ways that clinics and the broader public health care system shifted in order to meet the needs of this particular population, the analytic team explored a specific set of previously coded thematic areas. This manuscript represents findings from a subset of this previously coded data, exploring patterns from narratives related to changes to clinic procedures, staff burden, challenges experienced in HIV prevention and care, telehealth and remote work, and a category of findings we coded as “silver linings”—changes that resulted in better patient and staff experiences. A small team of analysts (EA, PD, NR) then read through narratives and met regularly for additional exploration and systematic analysis, particularly focusing on the data coded under each of the following three broad thematic areas: (1) impact of COVID on clinical systems, (2) workforce, and (3) access to care. We integrated key narrative passages into more detailed analytic memos, exploring patterns, contextualizing our understandings, and comparing data across the sites. The team met regularly throughout this process, discussing and further probing the data, often returning to the original transcripts. These understandings are summarized in the results below.

## Results

### Challenges to care engagement during COVID

Several challenges arose in the immediate aftermath of the public health emergency declared at the outset of the COVID pandemic in March 2020, which led to a constellation of impacts on the ability of people with HIV and SMI to access care. Leadership and provider perspectives on these impacts for both clinic and patient experiences are documented in subsections below.

### Loss of physical space

Initially, clinics transitioned to primarily telehealth-based modalities and in-person clinic programs that benefited vulnerable patients ceased. Shuttering physical spaces that provided waiting rooms and group meeting spaces, food pantries, social interaction, and access to other essentials posed important challenges to care engagement. One informant described how the loss of physical clinic space led to disengagement in care for men of color at his HIV clinic:

*“The morning was also pretty unique in that we had a breakfast club and… an education group for… men of color. They would come get a free meal… [During Shelter in Place] we lost the ability to get food for the enrollees in our men of color program. They stopped coming to clinic. We had to do some of the education stuff online, which didn’t work so well.” (KI05)*

Many behavioral health and HIV clinics also reported that the loss of space translated to a lost sense of community and social support for their patients. People were unable to physically congregate in one space for community and fellowship, which contributed to isolation, and in some cases, resulted in increased substance use among patients, as described by clinic administrators:

*“One day [each week], [patients] would come, and they would sit down, and they would hang out, and they [people with SMI] would get lunch and… it was a community. It was the only social interaction they had. I think the adverse [effect of the clinic closure] has… increased use of substances.” (KI03)*

*“And then the other sad thing has been our no-use space, which was a drop-in center for [clinic name] clients. So, people [with SMI] could drop in and meet with the peers and watch movies and make friends and eat lunch and all of those things. And, unfortunately, we haven’t reopened that quite yet because it was an environment where there’s a lot of coming and going and a lot of interactions and we just haven’t quite figured out how to safely resurrect that with COVID protocols.” (KI01)*

### Exacerbated mental health needs and disengagement in HIV care

Beyond the immediate impact of closed clinics and shifts to telehealth, COVID-19 also exacerbated existing mental health needs, which in some cases was associated with worse HIV outcomes. One HIV provider described the trends of HIV care disengagement in his clinic for patients with heightened mental health needs:

*“There are a couple of people who we just can’t seem to engage right now, probably because of worsening depression… They continue to struggle. We haven’t fully engaged some of my patients for whom I think that’s really an ongoing issue… Just sitting in your room. I’m like, get out your room, get some sun, go out. It was really hard on them.” (KI04)*

With symptom escalation in patients who already had mental health concerns, there was also an increase in avoidance behaviors tied to COVID-related fear and anxiety. One mental health provider discussed the relationship between avoidance and increases in patients with HIV withdrawing from behavioral health care:

*“I think, common to a lot of mental health conditions, we see one of the core features of mental health conditions being… avoidance. We actually saw a lot of people either drop out of care, not come to follow-up appointments, [be] lost to follow-up appointments… [we were] not sure where they were. And so, a lot of our patients also fell into that category where they may not necessarily have expressed an exacerbation or [worse expression] of symptoms, but actually avoided coming in altogether, withdrew from care, and that wasn’t an indicator that they were doing well. That was actually an indicator that they were actually suffering [more].” (KI05)*

Informants noted similar experiences regarding patient anxiety in attending in-person HIV care appointments during COVID, and even described how this anxiety contributed to increased HIV symptom burden and viral load:

*“There were definitely people who were afraid to come in, that kind of… fell out [of care]. Their HIV wasn’t well controlled. There [was also] a group that just wanted to [only] come in for their lab work, [which made] us really nervous. The clinic viral load went up during COVID.” (KI07)*

*“There’s a group of folks in my general practice who… definitely didn’t want to come in because of COVID… we had some issues there. For a fair number of people, I’m thinking 10–15%, it was bad, the isolation was not good for their [HIV health].” (KI04)*

One psychiatrist commented on how his clinic navigated these trends in HIV and behavioral health care disengagement as providers had the added burden of reviewing cases and reaching out to those who were falling through the cracks. This clinic took specific steps to maintain engagement in care:

*“Our clinic had always done a really good job of doing… consistent one-hour huddle[s] to… run through the list and see which patients [with SMI] are doing well, which patients are not doing well, which people are engaging in care, which people are not engaging in care. We took a very population health approach regardless of whether the people are coming in or not: we… ran through all the people to make sure that we were not having anyone fall through the cracks, and that interdisciplinary case conference… took place every Thursday morning.” (KI05)*

In sum, COVID not only reduced regular HIV and mental health care engagement and prevention, but it also impacted other forms of nonessential care, like access to social support and food pantries for additional nutritional support. While this was in part due to patient fears and closure of physical clinic spaces, as described above, the shift to telehealth also provided unique barriers that limited care engagement.

### Telehealth barriers and the digital divide

In clinics that were unable to offer patients phones or private in-clinic space for remote telehealth visits, informants emphasized that people faced several barriers to telehealth due to the digital divide. One psychiatrist working in an HIV prevention and treatment program summarized three major barriers his patients faced in accessing telehealth, namely (1) a lack of equipment, (2) no or limited internet, and (3) limited digital literacy:

*“I think…one [barrier] included not having either a computer, laptop, or an appropriate mobile device to be able to access Zoom. That was kind of one piece. I would say the second piece was not having appropriate internet, Wi-Fi, cellular service to be able to have that happen. And then, the third was…patient education around how to use the existing technology and also [their]… preference for using the existing technology.” (KI05)*

Due to these challenges, clinics began to find that their most vulnerable patients were difficult or impossible to locate. While telehealth was convenient for most, and led to more consistent overall engagement in care, hard-to-reach populations were often left behind due to lacking the appropriate resources to access telehealth. One HIV clinic director and provider worried about the long-term impact of COVID on vulnerable patients with HIV and mental health needs:

*“I think that although COVID in some ways expanded our potential capacity to widen our catchment area and provide more services, I think to our most vulnerable people, it may have actually widened the disparity, and I worry a lot about that. You don’t know what you don’t know. If these people have gone missing, you don’t know what befell them and why. I have a lot of anxiety about that, and that while [our institution], in particular, likes to pat itself on the back for how well it transitioned to remote services, I will be surprised if it was equally successful for all… On my personal panel, about 15 to 20 people seem to have disappeared and I can’t reach them. Either their phone is changed or disconnected.” (KI12)*

The challenges of the digital divide became more nuanced in behavioral health, as telehealth utilization was uniquely challenging for those with HIV and comorbid SMI. Specifically, the difficulty and complexity of navigating online systems was compounded by the need for in-person care. One psychiatrist working in an HIV outpatient clinic recalled the stressors and obstacles he faced in tracking down patients, particularly during critical situations when individuals needed help but were not on-site:

*“I still had to take care of these folks, and quite honestly, everyone’s in crisis. So, you know, I’m calling folks, documenting calls, and eventually, they like say, “Okay, you can actually bill for a telephone visit,” for pennies on the dollar. I’m chasing around folks who were suicidal and on methamphetamine, driving around the county. You know, things that I wouldn’t actually previously have had to deal with because they were in my office and I knew where they were, and I could even call emergency services if I needed to get them to the hospital. But now, I’m just like doing my best and documenting these really critical situations as best I can.” (KI09)*

### Burnout within the healthcare workforce

Informants across the dataset reported consistent stressors experienced by healthcare workers during the pandemic, which resulted in clinician and staff burnout. This ultimately resulted in significant turnover, leaving many clinics with both a provider shortage and heightened workload. The morale of the healthcare workforce was at a record low during the height of the pandemic: several healthcare workers faced the COVID-related deaths of both patients and colleagues while supporting family and other loved ones who became gravely ill. The clinic director of an HIV and behavioral health CBO described these overwhelming stressors:

*“Your workforce aren’t robots… they’re dealing with very intensive caseloads and issues that are real-life happening with the pandemic, and they have their own personal challenges, so not surprisingly, under those circumstances, [the workforce] would think twice about coming back.” (KI13)*

Given the changing circumstances of the pandemic, clinic staff and providers were working together and leveraging existing services in new ways to meet the needs of their patients. However, the workforce rarely received compensation for the additional labor. One physician in an HIV clinic described how these responsibilities were silently pushed onto providers, contributing to feelings of underappreciation and mistreatment.

*“One of those things that you get a lot of as a provider is “Don’t you care about your patients? Don’t you want to do this for them? Don’t you want to?”… The health system just tries to unload a lot of these innovations on our backs without really compensating… I think the health system either knowingly or unknowingly kind of took advantage of the goodwill of physicians to get a lot of that work done.” (KI09)*

### Silver linings: changes and adaptations that led to improved care

Silver linings were born out of the urgent need to change protocols due to the unprecedented nature of COVID. Providers rose to the complex and extraordinary challenges brought about by the burgeoning pandemic, adapting in stride to organizational, local, and state orders to ensure no lapses in patient care while simultaneously not unduly burdening patients or suspending services. Below we document several areas where clinics made changes that benefitted patients and staff.

### Improved health care delivery with telehealth

The COVID-19 pandemic forced clinics to embrace telehealth, and many clinicians and leaders described the pandemic as the necessary push for healthcare systems to finally take advantage of technology that had been underutilized prior to the pandemic. Here, a behavioral health clinic administrator described the role of the pandemic on telehealth:

“*I think when there’s a crisis, there’s an opportunity to do something [the shift to telehealth] that you’ve been wanting to do for 10 years, and suddenly, it magically appeared.*” (KI11)

As clinics implemented initial evaluations, consultations, medication management, pharmacotherapy, and psychotherapy remotely for the first time, one behavioral health provider and administrator marveled at both the significant changes and vast opportunities afforded through the shift to telehealth:

*“[Telehealth] was both miraculous and frustrating… our electronic health record was one that we could easily put on a virtual platform. …The department purchased licenses for this virtual desktop that pretty much everybody [had] access to. …We had a lot of educational sessions…for the physicians that [had] variable levels of digital competency. There was also a 24-hour…phone-in line for people that needed help with the EHR or with these different…electronic software systems.” (KI08)*

Telehealth encouraged integrated care and collaborative relationships between providers. Electronic health records allowed for widespread messaging to staff and providers, and video visits offered more accessible ways to deliver integrated mental health and HIV services, allowing multiple providers to share a visit with a patient as a team, as exemplified by one HIV provider:

*“I think we can dial in each other a little bit more. So, there have been times when I’ve had video visit, and [psychiatrist] can come in, or my pharmacist, and I think sometimes just allowing people to kind of share a visit with a patient. I’ve - I’ve done that with my geriatrician in our medical program. That also helps kind of make it easier for us to all be on the same page and the patient to feel like we’re all thinking about them.” (KI04)*

Two years after the initial adoption of telehealth, several informants reflected on the ways telehealth improved service delivery. Many have shifted their pre-pandemic views on telehealth, now believing it is “here to stay” as a new standard model for health care delivery:

*“We’re never going back to not being able to provide telehealth. Before, there was a lot of… uneasiness about being able to [successfully] provide telehealth services. Now, [it’s] just part of the model.” (KI13)*

### Increased care engagement with telehealth

Telehealth offered flexibility and convenience in providing care and meeting the needs of patients. As a result, many clinicians saw an uptick in care engagement and a decrease in no-shows. While telehealth changed the context and environment of visits (providers described patients taking phone visits “at the grocery store,” or “on the bus”), several informants observed that their clinics were ultimately able to reach more people, as telehealth offered the ability to cater to individual care delivery preferences and decrease traditional barriers to care (e.g., transportation). The convenience of telehealth visits also strengthened continuity of care and consistent provider-patient communication, as described by one psychiatrist and administrator of an HIV and behavioral health clinic:

*“[Patients] would be… at the grocery or… [at] another appointment. They were happy to [quickly] check-in on the phone and say, ‘yeah, everything’s fine, just give me my meds and I’m on my way.’” (KI02)*

Telehealth was especially effective and impactful for individuals with comorbid SMI who could navigate the digital health care system. These patients were physically more isolated during COVID, and struggled to attend in-person visits. This mental health provider described the ways remote visits were more comfortable for some of his comorbid SMI patients, stating that, *“some are just holed up at home, and they’re happy [not to have to attend in office visits].”* (KI02)

To ensure effective telehealth utilization for vulnerable patients, some clinics implemented creative strategies to minimize the digital divide by transforming physical clinic space for telehealth visits and providing clients with the proper technology. One informant described the tangible ways their HIV clinic addressed the digital divide:

*“Patients would still be able to kind of come into clinic… but they would be able to access their clinicians over the iPads that the clinic had set up and that the medical assistants helped to support so that they could access and talk to their clinicians whether that was a primary care clinician or a mental health provider or a pharmacist or one of the social workers even, and sort of have that seamless experience on the iPad.” (KI05)*

### New opportunities to provide services that help meet basic needs

Telehealth also allowed clinics the ability to leverage existing staff to provide novel continuity of care services not otherwise possible in an in-person setting. For example, one provider described utilizing their clinical pharmacy staff to approve medication refills:

*“If somebody were stable on medication, rather than coming into the clinic and meeting with the psychiatrist or even doing a virtual meeting, they could have medications refilled by clinical pharmacists [via] a phone intervention. They would do check-ins and then refill prescriptions, especially for those [people with SMI] who were at great risk. That helped…with continuity of care.” (KI08)*

This clinic also reported establishing a roving pharmacy team to administer COVID vaccinations in Los Angeles, as their prior presence in the community alleviated patient worries surrounding vaccines:

*“Our pharmacists are trained to [administer] vaccinations, so the [pharmacy] team moved from one location to another, vaccinating for maybe two days at [one] location, and then moving to a new location the [following] week. Six or seven clinical pharmacists… were the overseers of this… roving team, and said, ‘Hey, if [the Department of] Public Health will give us the injections, we will administer them. Some of our [patients with SMI]… they’re frightened. We were trusted caregivers already.” (KI08)*

The shift to remote work allowed many clinics to expand their scope of practice to also offer more wraparound services to patients. For example, one community health clinic began to offer ad-hoc social support services to patients to increase care engagement and promote stability during the pandemic. The clinic director described this shift:

*“Our home-based services definitely expanded. The teams [consist] of RN[s] and therapist[s]; they… [made] sure that people had groceries, reach[ed] out to schedule… appointments, [coordinated] Lyft rides [and] transportation, and [addressed] barriers. It was an extended conversation… [discussing] life issues [among] patients who didn’t have food [or] were afraid to go out.” (KI16)*

Through partnerships with local philanthropic organizations, another organization was able to meet basic needs by providing food services and financial support for clients:

*“Local philanthropic agencies were providing nutritional or food services [from] local food banks… and financial support… particularly during the peak of the epidemic, and a lot of our consumers were looking for that. Our clients are basically [saying], ‘We need this support.’ Being able to do that for our consumers …was definitely an added benefit.” (KI13)*

### Paradoxical impacts of COVID on HIV prevention and care

The COVID-19 pandemic shifted the HIV care landscape by revealing preexisting shortcomings in HIV care and prevention that had previously gone undetected. For example, changes in testing patterns and availability forced prevention programs and clinics to reflect about historical testing services and to consider new avenues for future improvements. Reconsidering outdated procedures and updating them became another area for possible improvement. One informant described inadequate HIV testing policies that were brought to light during COVID:

*“Our policies, up until just a few months ago, were pretty outdated. I would say that COVID had the impact of decreasing [HIV] testing overall when it was far below where it should have been even beforehand. People just weren’t coming into the clinics, because all the work was happening remotely.” (KI08)*

Unfortunately, in part due to delays in HIV testing, the pandemic may have worsened HIV and STI incidence rates within certain populations, though data on this is still emerging. For example, it is also possible that SIP meant for a reduction in higher risk activities. One HIV clinic director expressed concern about the long-term impacts of COVID on the HIV prevention landscape, noting patient-driven changes in behavior that may have led to disease transmission:

*“We had a lot of people… decide to come off PrEP during… the pandemic, which made me really anxious. It seemed… that some people were really good at assessing that they had made a conscious decision that they were just not going to go out and be [sexually] active. There were other people who were really bad at predicting that, and would end up sheltering in place with certain people and there was more sexual activity. I was very anxious that we were going to see a lot of seroconversions.” (KI12)*

*“As people have come out of COVID, we have seen an explosion… in gonorrhea, chlamydia, syphilis, multiple STIs, and repeatedly so. We don’t know the full story yet at a public health level, because… people are coming to identification and linkage of care now. We don’t know the full impact of [COVID] yet.” (KI12)*

While some providers worried about increased HIV cases, the pandemic positively impacted healthcare decision making at the patient level as well. This is paradoxical because COVID-related anxiety negatively impacted HIV care engagement for some patients, yet providers also witnessed the opposite effect: COVID fears encouraged other patients to re-engage with care and remain adherent to medication, as described by one HIV provider:

*“I could think of a couple of people who actually turned out to be really good, which is kind of paradoxical, because some of them really hunkered down. I can think of one guy we’d been trying for a long time to engage, two of them, and they were able to hunker down with some colleagues and friends who actually helped them organize their health care. And it’s like you’ve got to get this together. You can’t be sick [during] COVID. And they kind of got scared that they would die if they got COVID because they hadn’t been taking care of themselves. And so, actually, it paradoxically, it’s like very interesting how things happen for some people, right? I’m going to stay home, I’m going to take my meds, or I’m staying with some friends, I’m sheltering with some friends, and they just all just hunkered down. I can think of two very difficult [patients] in terms of controlling their HIV [that] just turned their lives around.” (KI04)*

### Instituting workforce wellness practices

One silver lining stemming from the exacerbated burden of trauma and grief faced by the healthcare workforce was increased awareness and de-stigmatization of workforce mental health needs. As a result, workforce wellness efforts were prioritized on an institutional level, and work culture changed to embrace self-care. One informant described this shift as inevitable, as healthcare workers had been *“put under this pressure cooker with…COVID.” (KI07)*. This leader described the addition of therapy for clinicians to help process the grief associated with COVID-related patient deaths:

*“We’re actually doing therapy - we have a facilitator that we started to work with back in the fall. We meet as a group on Zoom to process loss… what we go through as providers. It’s been extremely meaningful and beneficial.” (KI07)*

One clinic director noted the implementation of unit-specific staff development retreats, monthly staff development luncheons, and set-aside time for clinician emotional support during group supervision. This informant described the importance of integrating workforce wellness practices:

*“I think the ability to provide… ongoing self-care and professional development for our workforce is critical because, you know, staff burnout was already high prior to COVID and was certainly much higher during COVID. So, now integrating that as an ongoing practice for any behavioral health, HIV/AIDS organization is critical.” (KI13)*

This informant went on to point out the unique resilience of the HIV workforce, and the value of shared history for workforce social support:

*“I’ve learned that our organization and team is pretty resilient… it reminded me of the days of the AIDS epidemic when people were dying left and right… There’s a silver lining in that it’s not our first epidemic, and… because we have that muscle from the beginning of the AIDS epidemic, I really strongly believe that this made [COVID] a little bit easier, and reminding ourselves of our history that, you know, we’ve been in the trenches doing this [before].” (KI13)*

## Discussion

The COVID-19 pandemic radically impacted HIV and behavioral health care systems and service delivery in the United States. Clinics faced unprecedented challenges adhering to state and county mandates and safety guidelines, rapidly shifted to telehealth, and met changing patient and workforce needs. Across the board, providers and clinics reported establishing creative protocols at the outset of the pandemic to continue providing care to patients with co-morbid SMI and HIV by leveraging existing services in novel ways.

The mass transition to telehealth required a significant transformation of services and protocols; these transformations resulted in health care disparities between those with access to technology and those for whom access was more limited or challenging. Historically, delivering HIV care via telehealth delivery was uncommon: a Kaiser Family Foundation national survey of Ryan White providers found that, prior to the pandemic, only 22% offered telehealth services. Subsequently, however, over 99% delivered at least some HIV care and prevention through telehealth in the early months of the pandemic [13]. HIV clinics faced several obstacles to effective service delivery via telehealth, as some patients did not have access to appropriate devices, stable Wi-Fi for online video visits, or the necessary digital literacy to engage in online care. The logistical challenges patients faced led to dropout in care among some already hard-to-reach populations. These findings echo prior work on the digital divide during COVID-19, which describes the absence of technology, digital literacy, and reliable internet coverage as key barriers preventing effective telehealth [39]. These barriers ultimately served to prevent vulnerable populations (e.g., patients of color, older patients, patients with low socioeconomic status like people with SMI) from receiving HIV care during the pandemic [40–42]. Telehealth brought additional challenges in behavioral health for both patients with comorbid SMI and their mental health providers. In addition to digital accessibility issues, this population experienced burdens related to a lack of in-person support, especially for crisis intervention. In critical situations, behavioral health providers found it difficult to track down patients and felt a lack of control in their ability to provide care. These findings are in line with prior work on SMI patient and provider perspectives on telehealth during the pandemic, which note similar barriers to rapid, hands-on intervention [43]. 

The termination of in-person services ultimately exacerbated social isolation in PLWH, a population already experiencing higher rates of isolation in general [44]. Informants discussed the losses associated with clinic closures and loss of physical space. Prior to the pandemic, certain clinics provided hot meals and food pantries to populations engaged in HIV care and served as a space for community building and social support. This is also true of many public behavioral health clinics which provided not only food, but also a place of belonging for vulnerable groups with SMI. However, COVID-19 protocols forced many of these efforts to cease, and our informants reported that their patients and clients may have felt the adverse effects of this loss of consistent social interaction (e.g., isolation, increased substance use). This finding adds to Marziali, et al., which noted that organizations providing socialization opportunities for PLWH were required to limit non-essential programs as COVID-19 restrictions tightened.

Consistent with prior work, many providers interviewed reported an increase in mental health needs among patients with HIV. Informants recounted the effects of worsening mental health outcomes, like depression, which in turn, influenced patients’ ability to consistently engage in HIV care. Other commonly reported mental health symptoms included avoidance behaviors manifested through COVID anxiety, which in some cases, led to disengagement from care entirely. Multiple clinics discussed the various impacts of inconsistent HIV care, such as increased viral load, among patients who were anxious about the potential of contracting COVID during in-person visits. Clinics sought to combat these care engagement issues through creative approaches: some implemented stricter protocols with patient outreach, and others initiated weekly collaborative case conferencing with panel management to ensure continuity of care among their patients and loss to follow-up remained as minimal as possible. Paradoxically, COVID anxiety had a positive effect on HIV care in some cases, as providers saw patients re-engage in care due to fear surrounding increased susceptibility to COVID-19. Similar behaviors were reported in a recent study from New York, where some previously lost patients similarly re-engaged during COVID [45]. 

Across all settings, the HIV and behavioral healthcare workforce faced a steep learning curve with constantly changing protocols as clinics navigated the pandemic in real time. This contributed to burnout, higher staff turnover, and low morale, in conjunction with the emotional and physical burden of intensive caseloads, patient mortality, increases in viral load, and feelings of general underappreciation. In response to considerable clinician and staff burnout, some HIV and behavioral healthcare clinic directors introduced practices and resources to shift unhealthy workforce culture and foster workforce wellness in this provider population. For example, one clinic implemented group therapy sessions for clinicians, which allowed them a collective environment in which they could process grief and trauma surrounding COVID- or HIV-related morbidity and mortality of patients, colleagues, and loved ones. Another clinic instituted staff retreats and luncheons, which helped staff simultaneously prioritize self-care and professional development. These practices could be used in addition to existing research on healthcare workforce wellness [46, 47], organizational changes prioritizing employee interests strengthen commitment to work, and the availability of collaborative environments during the pandemic that increased support and camaraderie among staff [48]. One HIV clinic director contextualized this finding, noting that COVID-related camaraderie within the HIV workforce was reminiscent of the resilience built in the face of the HIV epidemic.

While several challenges brought about by the COVID pandemic were detrimental to patient services and outcomes, several other changes brought about increases in care engagement and greater overall treatment success. On the patient side, consumers were provided a novel method by which to access healthcare that did not require in-person visits to receive services. This proved more comfortable for many patients, especially those with HIV and SMI, and contributed to the perception of increased care engagement and reduced no-show rates, which is consistent with prior findings on decreased nonattendance [49] and positive patient responses to telehealth in HIV care [50]. In cases where patients were willing and able to attend office visits, they were often afforded increased service availability and the opportunity to receive vaccinations, food and other basic needs at healthcare appointments. On the provider side, the widespread adoption of telehealth was facilitated not only by funding to set up the necessary infrastructure, but also by new abilities for providers to bill insurance providers for remote behavioral health and HIV care visits. As provider efforts to expand telehealth were met with resistance for years prior to the pandemic in these domains, as well as within healthcare more broadly [51], COVID left the US health care system no choice but to lean into the provision of remote services. Telehealth allowed providers to collaborate more seamlessly through EHR and shared video visits with patients. This promoted integrated care and resulted in greater flexibility for providers to tailor care to individual patients, increasing care engagement and success overall.

There are several strengths and limitations to consider. We intentionally chose a sample of key informants who remained engaged in providing services to those impacted by HIV and SMI, therefore we did not include staff or providers who burned out and left the workforce in the wake of COVID. Similarly, our study focused on changes that were made in response to the COVID pandemic and shelter in place orders that were instituted in the State of California, where the Governor issued statewide lockdown orders that continued for many months. Other settings, including those in low to middle income countries, may not have experienced the same kinds of public health mandates during the COVID pandemic that were instituted in the California setting and therefore may have had different kinds of resources and outcomes with their patient populations and care engagement. Another limitation is that informants did not include patients, and so descriptions of patient experiences are all based on observations from their providers and clinic administrators. Still, we believe that the insights that our key informants provided offer some important perspectives on creative solutions to engaging this patient population in care, maintaining connections to both HIV and behavioral health services.

### Conclusions and implications for practice

Although the COVID pandemic forced many clinics and providers to quickly shift protocols, maintaining operations under immense pressures, several of the changes benefitted this unique patient population with comorbid HIV and SMI, as well as the providers and staff who care for them. These silver linings can inform strategies to support this population in future pandemics. Strategies include (1) helping patients with comorbid HIV and SMI remain engaged by creating flexible ways to connect to care through telehealth, which allows multiple providers to collaborate more conveniently and provide integrated care for patients; (2) expanding the roles of allied health professionals (e.g., to refill prescriptions or deliver vaccinations); (3) instituting workforce wellness practices, such as opportunities to process grief and receive validation during periods of high stress, which allows those on the frontlines to heal and remain engaged in their work, avoiding burnout; (4) enhancing the wellbeing of this particular patient population by attending to patients in a holistic manner, recognizing the importance of fulfilling basic needs such as food and shelter, and providing social opportunities for isolated patients where possible. Finally, learning to be responsive and adaptive is an important lesson as healthcare providers move ahead and confront emerging challenges (e.g. environmental, emerging infectious diseases, etc.) and can lead to better pandemic preparedness for the future.

### Electronic supplementary material

Below is the link to the electronic supplementary material.

Supplementary Material 1

## Data Availability

The datasets generated and/or analyzed during the current study are not publicly available due to privacy protection of participants. Please contact Emily Arnold (emily.arnold@ucsf.edu).

## Abbreviations

SMI: Severe mental illness
CBO: Community-based organization
SIP: Shelter-in-place
CDC: Centers for Disease Control and Prevention
PLWH: People living with HIV
SFDPH: San Francisco Department of Public Health
PrEP: Pre-exposure prophylaxis
