# Supplementary material for: Care challenges and silver linings in HIV and behavioral health service delivery for individuals living with HIV and severe mental illness during the COVID-19 pandemic: a qualitative study
Source: BMC Health Serv Res. 2024 May 31;24:690. doi: 10.1186/s12913-024-11146-1 (PMC11143645; doi:10.1186/s12913-024-11146-1)
Supplement: Supplementary file 1 — Supplementary Material 1 [file 12913_2024_11146_MOESM1_ESM.docx]

**Appendix A: Interview Guide for Impact of COVID on clinics serving HIV/SMI populations**

**Tell me a little bit about your role in your clinic.**

How long have you been in this role? What led you into this line of work?

**What kinds of services do you provide at your clinic?** How are those tailored for people with HIV? SMI? What % of caseload are with SMI?

**Thinking back to two years ago, can you tell me a little bit about how your clinic was impacted by your clinic was impacted by the COVID pandemic.**

What policies were put in place?

How were clinic procedures and services changed as a result of these policies?

**Please describe efforts to ensure access to HIV testing and PrEP/PEP during COVID-19**

- - Were there any efforts specifically targeting people with severe mental illness?

Were there any barriers to implementation? Could you characterize the levels of these barriers and how they might be overcome?

**Please describe efforts to maintain access to HIV care and treatment during COVID-19**

- - Were there any efforts specifically targeting people with severe mental illness?
  - Were there any barriers to implementation? Could you characterize the levels of these barriers and how they might be overcome?

**Please describe the degree to your clinic tried to maintain access to mental health services during COVID.**

- - To what degree are MH services integrated into existing services you offer at the clinic? How has this evolved over time?
  - Have you seen additional needs for mental health services in HIV settings with the emergence of the COVID pandemic and resulting public health policies?
  - Are there any programs that are particularly notable for achieving well-integrated care, and that have continued to be engaged with their patients during this time?
  - How has telehealth featured in the ability to provide robust services to individuals affected by HIV and SMI during this time? What has worked? What could be better?

Slightly shifting gears, we have heard that electronic medical records can sometimes present barriers to providing well integrated care for those with complex care needs. Can you **Describe electronic medical data system(s) in place for mental health and HIV-related care**

# How does this system influence HIV care and service integration?

- - What are current practices around data sharing between behavioral health providers and HIV specialists? Are there any privacy concerns about this data sharing?
  - Were there any changes made to electronic data sharing agreements or policies during the COVID era?

Is there anything else you think we should know about in considering the impact of COVID on maintaining services to those with HIV and mental health needs?

Anyone else we should talk to?
